# Four days of bed rest increases intrinsic mitochondrial respiratory capacity in young healthy males

**DOI:** 10.14814/phy2.13793

**Published:** 2018-09-17

**Authors:** Steen Larsen, Anne‐Kristine M. Lundby, Sune Dandanell, Laura Oberholzer, Stefanie Keiser, Andreas B. Andersen, Thomas Haider, Carsten Lundby

**Affiliations:** ^1^ Xlab Center for Healthy Aging Department of Biomedical Sciences Faculty of Health Sciences University of Copenhagen Copenhagen Denmark; ^2^ Clinical Research Centre Medical University of Bialystok Bialystok Poland; ^3^ Institute of Physiology University of Zürich Zürich Switzerland

**Keywords:** Bed rest, glucose tolerance, mitochondria, reactive oxygen species production

## Abstract

Bed rest leads to impaired glucose tolerance. Whether this is linked to maladaptation's in skeletal muscle mitochondrial function and in particular to the level of reactive oxygen species (ROS) is at present unknown. The aim of this longitudinal study was to quantify skeletal muscle mitochondrial function (respiratory capacity and ROS production) together with glucose tolerance after 4 days of strict bed rest in healthy young male subjects (*n *=* *14). Mitochondrial function was determined in permeabilized muscle fibers using high‐resolution respirometry and fluorometry, mitochondrial content (citrate synthase [CS] activity) and antioxidant protein expression levels were assessed in parallel to this. Glucose tolerance was determined by means of oral glucose tolerance tests. Intrinsic mitochondrial respiratory capacity was augmented after the bed rest period (CI + II_*P*_: 0.43 ± 0.12 vs. 0.55 ± 0.14 [pmol/sec/mg]/CS activity), due to a decreased CS activity (158 ± 39 vs. 129 ± 25 mU/mg dw.). No differences were observed in ROS production (per mg of tissue or when normalized to CS activity). Furthermore, the protein content for catalase was increased while superoxide dismutase and glutathione peroxidase remained unaffected. These findings were accompanied by an impaired glucose tolerance after the bed rest period (Matsuda index: 12 ± 6 vs. 9 ± 5). The change in intrinsic mitochondrial respiratory capacity could be an early indication in the development of impaired glucose tolerance. The increased catalase protein content might explain that no change was seen in ROS production after 4 days of bed rest. Whether these findings can be extrapolated to lifestyle‐dependent decrements in physical activity and the development of type‐2‐diabetes remains unknown.

## Introduction

Physical inactivity is a major risk factor for type‐2‐diabetes (T2D) (LaMonte et al. 1985). Similarly, glucose tolerance impairments and reduced insulin sensitivity are observed after short and long periods of bed rest in healthy individuals (Dandanell et al. 1985; Alibegovic et al. 2009; Dirks et al. 2014). Such bed rest periods are of relevance for hospitalized patients, in Denmark in 2012 the average hospital admission was 4 days. Since impaired glucose tolerance is mostly considered a skeletal muscle disease, a number of local regulating mechanisms have been proposed important such as a decreased skeletal muscle glycogen synthase activity and glucose transporter 4 (Glut 4) capacity (Bienso et al. 2012) but nonetheless remain inconclusive regarding their respective importance. In recent years, reduced skeletal muscle mitochondrial content and function (intrinsic mitochondrial respiratory capacity and reactive oxygen species production) has been proposed important in the development of T2D (Mogensen et al. 2007; Phielix et al. 2010) although this remains debated (Larsen et al. 2009, 2011; Hey‐Mogensen et al. 2010). Regardless, the regulating mechanisms that could alter mitochondrial function with physical inactivity such as bed rest remain obscure. Skeletal muscle mitochondrial reactive oxygen species (ROS) production could be mechanistically involved as mitochondrial superoxide production blocks three important pathways of hyperglycemic damage (Nishikawa et al. 2000). This however has not been investigated with regard to impaired glucose tolerance in humans. It has been reported that ROS production tends to be increased in patients with type 2 diabetes and that physical activity rescues this to some extend (Hey‐Mogensen et al. 2010). Furthermore, excess intake of fat in the diet which is known to induce insulin resistance leads to increased ROS production in the skeletal muscle (Anderson et al. 2009). Therefore it is appealing to determine the potential relation between bed rest, impaired glucose tolerance, mitochondrial respiratory capacity and ROS production. Another potentially important mitochondrial related factor for glucose tolerance is the balance between mitochondrial fission and fusion which is important for the maintenance and operation of the mitochondrial network. Whether this is altered with physical activity or inactivity (bed rest) also remains unexplored. It has been reported that mitochondrial dynamics (fusion and fission) is affected in patients with type 2 diabetes (Liesa et al. 2009; Zorzano et al. 2009).

In this study, we took the opportunity to investigate the effects of 4 days of strict bed rest on mitochondrial respiratory capacity and ROS production in otherwise healthy young subjects. We hypothesized that mitochondrial respiratory capacity and content would be similar with only 4 days of bed rest, but that ROS production would be increased.

## Materials and Methods

### Ethical approval

The study was performed in accordance with the standards set by the Helsinki declaration, and was approved by the research Ethics Committee of Zürich, Switzerland (KEK‐ZH‐Nr. 2015‐0453), (Clinical trial number: NCT02612597 at http://www.clinicaltrials.gov). Subjects were recruited and the study was conducted early 2016. All subjects were informed orally and in writing about the experiments and potential risks before written consent was obtained.

### Subjects and bed rest

Some of the data has been published as part of a study investigating the effects of blood volume changes on glucose tolerance during bed rest (Dandanell et al. 1985) where fifteen subjects were included. Fourteen healthy normal glucose tolerant male volunteers (Age: 24 ± 4 years, weight: 75 ± 5 kg, BMI: 23 ± 1 kg × m^−2^; mean ± SD) participated in this study (mitochondrial analysis was missing on one subjects that is why only fourteen subjects were included in this study), subject characteristics are given in Table 1. The subjects were placed in hospital beds with manual elevation during the 4 days of bed rest and were under surveillance at all times. The subjects entered bed rest immediately after the premeasurements were finished and remained in bed for four complete days (96 h). Postmeasurements were performed before the subjects moved out of their beds. For more details see (Dandanell et al. 1985). The 4 days of bed rest was selected because the average hospital admission in Denmark in 2012 was 3.7 days.

**Table 1:** Subjects characteristics (*n *=* *14) before and after bed rest

	Pre	Post
Body weight (kg)	75 ± 5	75 ± 5
Total lean body mass (kg)	58 ± 4	58 ± 4
Total fat (%)	18 ± 6	19 ± 6
VO_2max_ (mL/min)	3371 ± 557	3330 ± 408
VO_2max_ (mL/min/kg)	45 ± 7	45 ± 5
Fasting glucose (mmol/L)	4.9 ± 0.3	4.9 ± 0.2
Fasting insulin (pmol/L)	30 ± 32	39 ± 23
Glucose, 120 min (mmol/L)	5.3 ± 1.1	5.7 ± 1.4
Insulin, 120 min (pmol/L)	135 ± 85	234 ± 171^a^
Glucose AUC (mmol/L/min)	146 ± 55	209 ± 97^a^
Insulin AUC (pmo/L/min)	3906 ± 1373	5254 ± 2648^a^
Matsuda‐index	12 ± 6	9 ± 5^a^

Data are means ± SD. AUC, area under the curve; VO_2max_, maximal oxygen uptake.

a *P *< 0.05.

A limitation to the study is the lack of a control group, in order to see the changes in ROS production and mitochondrial respiratory capacity after 4 days. Unpublished data from the group have found no changes in these parameters over 2 weeks (authors own observation).

### Skeletal muscle biopsies

Skeletal muscle biopsies were obtained under standardized conditions from the *vastus lateralis* muscle on the day the subjects entered the bed rest and again after 4 days before leaving the bed. Samples were collected under local anesthesia (1% lidocaine) of the skin and superficial muscle fascia, using the Bergström technique (Bergström et al. 1967) with a needle modified for suction. The biopsy (100–200 mg) was immediately dissected free of fat and connective tissue and divided into sections for further processing: (1) high‐ resolution respirometry and fluoremetry measurements, and (2) enzyme activity and protein expression (snap‐frozen in liquid nitrogen). All samples were labeled with codes unknown to the investigator performing the analysis.

### Mitochondrial respiration and respiratory protocol

Mitochondrial respiratory capacity and electron transfer system capacity were measured in permabilized muscle fibers (PFi) applying procedures and conditions that previously have been described in details (Jacobs et al. 2013).

The following protocol was applied on PFi in buffer Z (Perry et al. 2013) after addition of 25 *μ*mol/L blebbistatin (Perry et al. 2011): Malate (2 mmol/L), octanoyl carnitine (250 *μ*mol/L) (ETF_*L*_) and ADP (5 mmol/L) (fatty acid linked respiratory capacity (ETF_*P*_). Subsequently, pyruvate (5 mmol/L) and glutamate (10 mmol/L) were added (complex I linked respiratory capacity, CI_*P*_) followed by succinate (20 mmol/L) (complex I and II linked respiratory capacity, CI + II_*P*_). Next, cytochrome c (10 *μ*mol/L) was added to control for outer mitochondrial membrane integrity and finally FCCP was titrated in 0.5 *μ*mol/L steps to assess uncoupled respiration. If mitochondrial respiratory capacity increased more than 10% after addition of cytochrome c the experiments was not used in the analysis. We did not remove any experiments due to high cytochrome c response. The ratio between cytochrome c and CI + II_*P*_ was 1.02 ± 0.06 before bed rest and 0.99 ± 0.04 after bed rest indicating that the mitochondrial preparation was reliable.

### Mitochondrial H_2_O_2_ production

The O2k‐Fluorometer (OROBOROS O2k‐Fluorometer, Oroboros, Austria) was used for simultaneous measurement of O_2_ consumption and H_2_O_2_ release in PFi. The H_2_O_2_ release can be measured with Amplex red in the presence of horseradish peroxidase. H_2_O_2_ reacts with Amplex red in a 1:1 relationship to form resorufin, a stable fluorescent compound. Addition of superoxide dismutase (SOD) will convert released superoxide into H_2_O_2_. Resorufin production is detected as an increase in fluorescence intensity and the time derivative equals the fluorescence release (ΔF × s^−1^). By including calibration steps in the protocol, it is possible to convert ΔF × s^−1^ into pmol H_2_O_2_ × s^−1^.

The following protocol was applied on PFi in buffer Z after the addition of 25 *μ*mol/L blebbistatin (Perry et al. 2011): Amplex Red (5 mmol/L, Molecular probes), Superoxide Dismutase (45 U/mL, Sigma), Horseradish Peroxidase (6 U/mL, Sigma) for establishment of baseline fluorescence signal. Sequential substrate addition was always flanked by titrations of freshly made H_2_O_2_ (100 nmol/L aliquots) for establishment of standard curves for each measuring condition: 2 × H_2_O_2_, followed by malate (2 mmol/L) and pyruvate (5 mmol/L) leading to CI leak. Then H_2_O_2_ (100 nmol/L), followed by three titrations of succinate (1 mmol/L) (nonsaturated CII leak), H_2_O_2_ (100 nmol/L), succinate (additional 2 mmol/L [3 mmol/L in total]) (nonsaturated CII leak), H_2_O_2_ (100 nmol/L), succinate (additional 7 mmol/L [10 mmol/L in total]) (saturated CII leak). This titration of succinate gives the opportunity to investigate the sensitivity for succinate. Finally, H_2_O_2_ (100 nmol/L), ADP (5 mmol/L) and times H_2_O_2_ (100 nmol/L) was added. For the analysis background fluorescence was subtracted and the standard curve established for each measuring condition was used for the calculation of H_2_O_2_ production.

### Muscle Lysate Preparation

Snap frozen muscle biopsy samples with an average weight of 15 mg were freeze‐dried overnight (ScanVac, Denmark). Next day, the freeze dried muscles pieces (average weight 4 mg) were homogenized (Precellys24Tissue Homogenizer, Bertin Technologies) in a fresh batch of homogenization buffer containing: 10% glycerol, 20 mmol/L sodium‐pyrophosphate, 150 mmol/L NaCl, 50 mmol/L HEPES, 1% NP‐40, 20 mmol/L *β*‐glycerophosphate, 2 mmol/L Na_3_VO_4_, 10 mmol/L NaF, 2 mmol/L phenylmethanesulfonyl fluoride, 1 mmol/L EDTA, 1 mmol/L EGTA, 10 *μ*g × mL^−1^ aprotinin, 10 *μ*g × mL^−1^ leupeptin, and 3 mmol/L benzamidine in a 1 mg/80 *μ*l ratio. Samples were then rotated end over end for 1 h at 4°C and centrifuged at 16,500 *g* for 30 min at 4°C and the lysates were divided into aliquots and stored at −80°C until further analysis. Total protein concentrations were determined by BCA assay (Pierce, Rockford, IL).

### Citrate synthase

Citrate synthase (CS) activity was assayed in muscle lysates using a commercially available CS assay kit (C3260, Sigma‐Aldrich) according to the manufacturer's instructions. CS activity was normalized to mg of total protein.

### Western blotting

Muscle lysates were diluted to the same protein concentration in Laemmli buffer (Bio‐Rad Laboratories) and heated for 10 min at 80°C. 10 *μ*g of protein was loaded on 15–4% Criterion TGX Stain‐Free polyacrylamide SDS gels (Bio‐Rad) and separated by SDS‐PAGE (Criterion Cell, Bio‐Rad). The gels were activated with ultraviolet light for 5 min and imaged for 3 s (Gel Doc XR system, Bio‐rad) before transfer to PVDF (polyvinylidene fluoride) membranes (0.2 *μ*m pores, Bio‐Rad), using the Trans‐Blot Turbo Transfer system (Bio‐Rad). After the transfer, the membranes were imaged with UV light for 3 s to quantify the protein transfer. The membranes were blocked for 1 h at room temperature with either skimmed milk or BSA diluted in Tris‐buffered saline (10 mmol/L Tris Base, 150 mmol/L NaCl, pH 7.4) + 0.05% Tween, and incubated overnight at 4°C with mitofusin2 (MFN2) monoclonal antibody (9927‐M03, Abnova) diluted 1:500 in PBS‐T 3% BSA, catalase monoclonal AB (ab16731, Abcam) diluted 1:2000 in TBS‐T 5% skim milk, superoxide dismutase 2 monoclonal AB (SOD2) (ab13534, Abcam) diluted 1:1000 in TBS‐T 5% skim milk or glutathione peroxidase 1 (GPX1) monoclonal AB (3286, Cell Signaling) diluted 1:500 in TBS‐T 5% BSA. One hour incubation at RT with secondary anti‐mouse antibody (1:5000) conjugated to horseradish peroxidase (W4021, Promega) was performed the next day. Bands were detected with Luminatea™ Classico (EMD Millipore) and imaged on an ImageQuant LAS 4000 (Fujifilm; Life Science). Band intensities were quantified using Image J (http://imagej.nih.gov/ij/, 1997–2014) and determined as the total band intensity minus the background intensity. Primary antibodies were optimized by use of human muscle lysates to assure that the amount of loaded protein would result in band signal intensities within the linear part of a standard curve. Pre‐ and postsamples were loaded on the same gel. Signal intensity from each muscle sample was adjusted according to the mean signal intensity of all samples on the same gel and relative to total Stain‐Free fluorescence of the single lane.

### Statistics

The statistical analysis was performed in GraphPad Prism 6. *P *< 0.05 was considered statistically significant. Data are presented as means ± SD unless stated otherwise. All data were evaluated for normal distribution and equal variance. CS activity, protein content, different mitochondrial ratios (pre and postbed rest) were compared, using a paired *t*‐test. Mitochondrial respiratory capacity and ROS production measurements were compared, using a two‐way ANOVA with repeated measures. Pearson′s correlation analysis was performed to establish the presence of correlations. All figures were made using GraphPad Prism 6 software (La Jolla, CA). Power calculations were performed prior to the study started and 12 subjects were found to be enough when mitochondrial respiratory capacity and reactive oxygen species production was investigated.

## Results

Subject characteristics and glucose homeostasis have been presented previously (Dandanell et al. 1985) where fifteen subjects were included. Briefly no difference was found in lean body mass or fat mass, and maximal oxygen uptake was also similar after bed rest. Area under the curve for plasma glucose and insulin concentration during an oral glucose tolerance test (OGTT) was elevated after bedrest (Dandanell et al. 1985). Furthermore, when calculating the Matsuda index for glucose tolerance, a decreased tolerance was found after the bed rest period (Table 1).

### Mitochondrial characteristics

**Figure 1:**
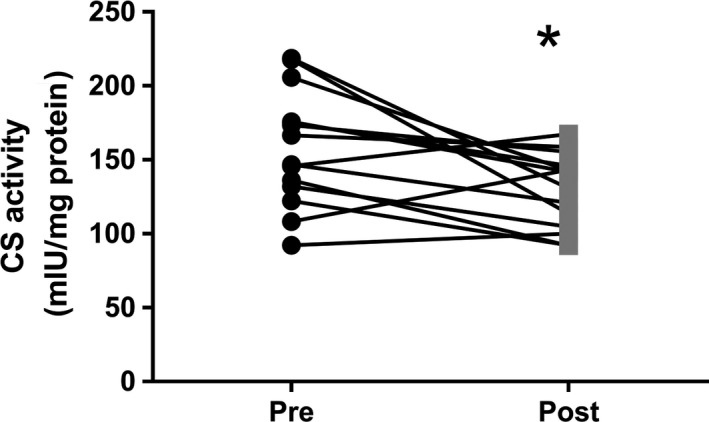
Citrate synthase activity. Data are means ± SD. *P <* *0.05

**Figure 2:**
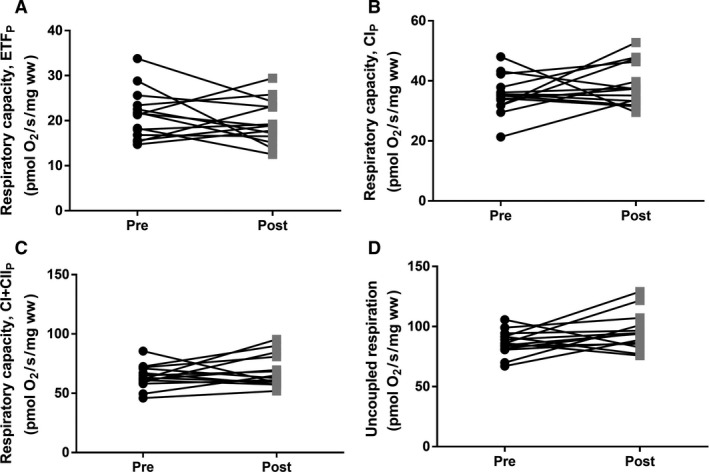
Mitochondrial respiratory capacity before and after 4 days bed rest. (A) Maximal mitochondrial lipid respiratory capacity (ETF_*P*_). (B) Mitochondrial respiratory capacity with complex I linked substrates (CI_*P*_). (C) Maximal mitochondrial respiratory capacity with complex I‐ and II‐linked substrates (CI + II_*P*_) (D) Uncoupled respiration. Data are mean ± SD. Black circles represents prebed rest; gray squares represents postbed rest.

**Figure 3:**
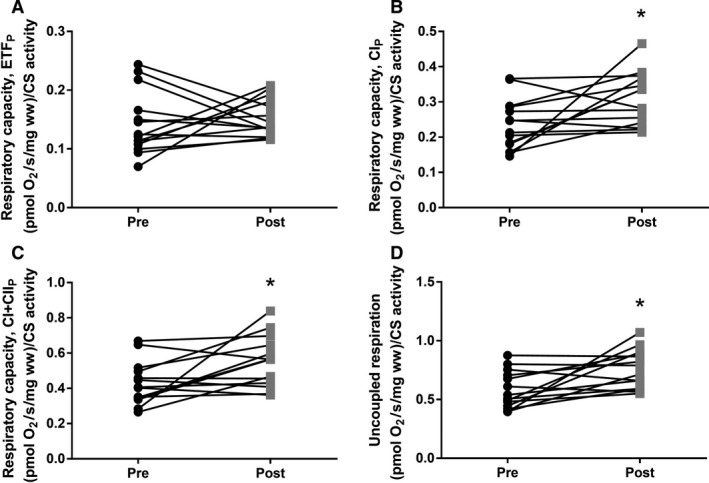
Intrinsic mitochondrial respiratory capacity (mitochondrial respiratory capacity normalized to Citrate Synthase activity) before and after 4 days bed rest. (A) Maximal mitochondrial lipid respiratory capacity (ETF_*P*_). (B) Mitochondrial respiratory capacity with complex I linked substrates (CI_*P*_). (C) Maximal mitochondrial respiratory capacity with complex I‐ and II‐linked substrates (CI + II_*P*_) (D) Uncoupled respiration. Data are means ± SD. *P < 0.05. Black circles represents prebed rest; gray squares represents postbed rest.

**Figure 4:**
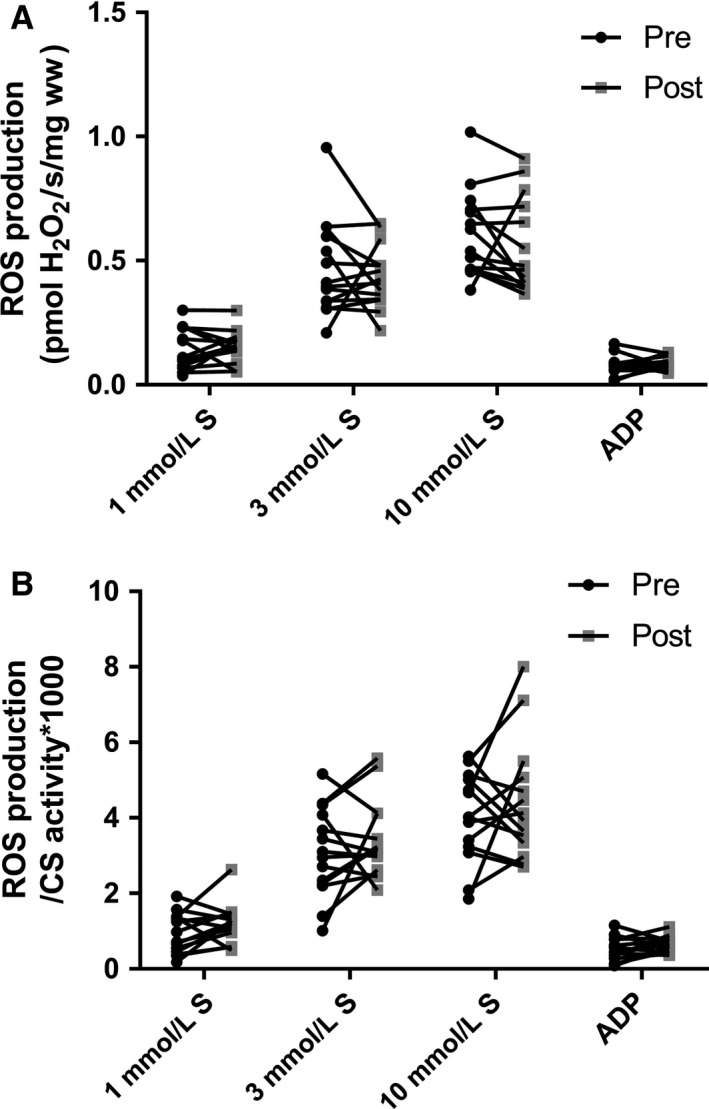
Mitochondrial H_2_O_2_ production before and after 4 days bed rest. (A) Succinate (1, 3 and 10 mmol/L) and ADP (5 mmol/L) induced H_2_O_2_ production in permeabilized muscle fibers (B) H_2_O_2_ production from panel A normalized to CS activity. Data are mean ± SD. Black circles represents pre bed rest; gray squares represents post bed rest.

**Figure 5:**
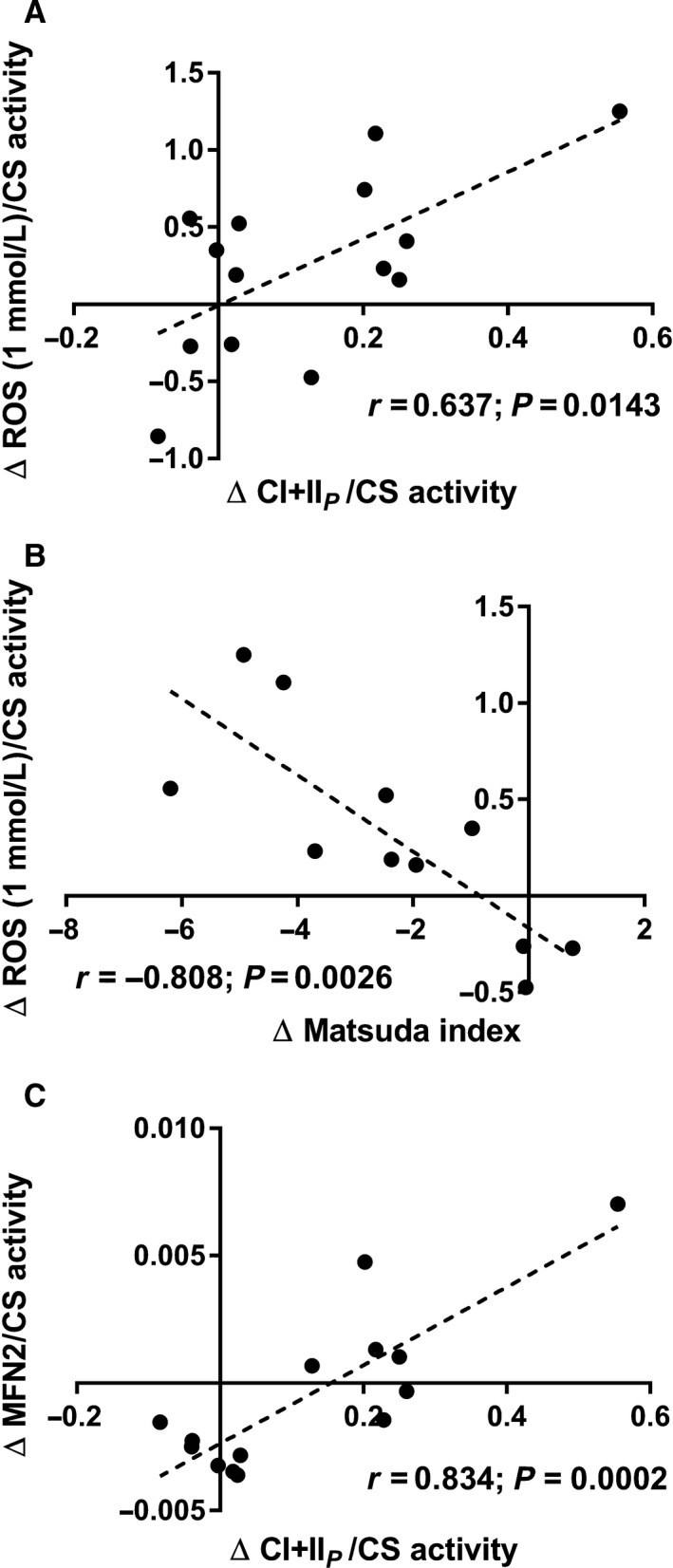
(A) Correlation between changes in mitochondrial respiratory capacity (CI + II_*P*_) and ROS production (1 mmol/L succinate) both normalized to CS activity. (B) Correlation between changes in the Matsuda index and ROS production (1 mmol/L succinate) normalized to CS activity. (C) Correlation between changes in mitochondrial respiratory capacity (CI + II_*P*_) and MFN2 both normalized to CS activity.

CS activity was decreased after bedrest (*P *=* *0.014) (Fig. 1), whereas no differences were found in mitochondrial respiratory capacity (normalized to wet weight) after bedrest regardless of substrate combination (Fig. 2). In contrast hereto intrinsic mitochondrial respiratory capacity (mitochondrial respiratory capacity normalized to CS activity) was increased after bedrest with complex I linked substrates, complex I + II linked substrates and uncoupled respiration (Fig. 3). Decreased ETF_*P*_/CI_*P*_ and ETF_*P*_/CI + II_*P*_ were observed after bedrest, whereas no changes were noted for ETF_*L*_/ETF_*P*_ and CI + II_*P*_/ uncoupled respiration (Table 2). ROS production did neither change after the bed rest period in absolute numbers (Fig. 4A) nor when ROS production was normalized to CS activity (Fig. 4B). A correlation was observed between changes in mitochondrial respiratory capacity (CI + II_*P*_) and ROS production (1 mmol/L) when both were normalized to CS activity (*r *=* *0.637; *P *=* *0.0143) (Fig. 5A). The changes in the Matsuda index correlated negatively with the changes in ROS production (1 mmol/L) when it was normalized to CS activity (*r *=* *−0808; *P *=* *0.0026) (Fig. 5B).

**Table 2:** Mitochondrial ratios

	Pre	Post
ETF_*L*_/ETF_*P*_	0.45 ± 0.13	0.44 ± 0.11
ETF_*L*_/CI + II_*P*_	0.15 ± 0.04	0.13 ± 0.03^a^
CI + II_*P*_/uncoupled respiration	0.75 ± 0.09	0.71 ± 0.11
ETF_*P*_/CI_*P*_	0.60 ± 0.13	0.51 ± 0.09^a^
ETF_*P*_/CI + II_*P*_	0.33 ± 0.07	0.29 ± 0.06^a^

All the ratios presented in the table are derived from mitochondrial respiratory data. Data are means ± SD. CI_*P*_, Malate, octanoyl carnitine, ADP, pyruvate & glutamate; CI + II_*P*_, Malate, octanoyl carnitine, ADP, pyruvate, glutamate & succinate; ETF_*L*_, Malate & octanoyl carnitine; ETF_*P*_, Malate, octanoyl carnitine & ADP; Uncoupled respiration, Addition of FCCP.

a *P *< 0.05.

**Figure 6:**
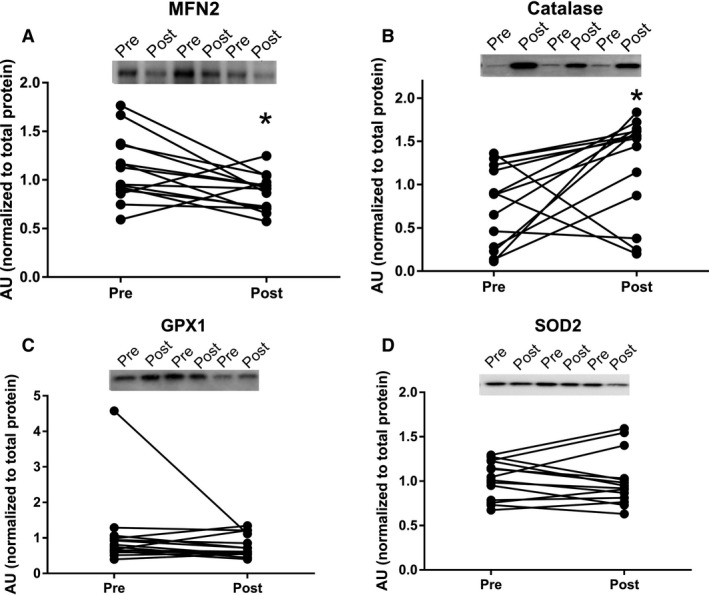
Protein expression levels before and after 4 days bed rest, a representative blot is shown on each figure. Data are means ± SD. *P < 0.05. Black circles represents prebed rest; gray squares represents postbed rest. GPX1 indicates glutathione peroxidase 1; MFN2, mitofusin‐2; SOD, superoxide dismutase.

A decreased MFN2 content whereas an increased catalase content was observed after the bed rest, but no difference was seen for the other antioxidants (superoxide dismutase [SOD2] or glutathione peroxidase 1 [GPX1]) after bed rest (Fig. 6). The change in mitochondrial respiratory capacity (CI + IIP) correlated strongly with the change in MFN2, both normalized to mitochondrial content (CS activity) (*r *=* *0.834; *P *=* *0.0002) (Fig. 5C).

## Discussion

The novel finding in this study was that 4 days of bed rest lead to an increased intrinsic mitochondrial respiratory capacity combined with a decrease in MFN2. While no differences were observed in ROS production (per mg of tissue or when normalized to CS activity). At the same time, the catalase protein content was increased, with no changes observed in superoxide dismutase and glutathione peroxidase. Further, changes in intrinsic respiratory capacity correlated with changes in ROS production (normalized to CS activity). These mitochondrial changes might be an early indication in the development of impaired glucose tolerance, as was seen in this study.

A reduction in CS activity was seen after 4 days of bed rest in this study, which is in agreement with another study applying strict bed rest for 7 days (Dirks et al. 2016). Similar findings have been reported when applying immobilization for 14 days as a model to reduce physical activity (Abadi et al. 2009; Gram et al. 2014). This indicates that mitochondrial content is likely reduced after a short period of bedrest (Larsen et al. 2012). Mitochondrial respiratory capacity (normalized to wet weight) was also investigated in this study, and did not change after bed rest. This is contradictory to the study by Gram and colleagues where a reduction in mitochondrial respiratory capacity was seen after 14 days of immobilization (Gram et al. 2014). This could however be caused by the very different interventions applied, where 14 days of immobilization was used by Gram and colleagues compared to 4 days of strict bed rest in this study. Although a decreased ratio between ETF_*P*_ and CI_*P*_ and CI + II_*P*_ linked respiratory capacity was observed, no change was seen in ETF_*P*_ indicating that the contribution from lipid oxidation are not affected after bed rest. An increased intrinsic mitochondrial respiratory capacity was found when complex I linked, complex I + II linked substrates, and uncoupled respiration were investigated. Intrinsic mitochondrial respiratory capacity has to our knowledge not been determined previously with regard to bed rest, although Gram and colleagues found no changes after 2 weeks of immobilization (Gram et al. 2014). It has been reported that intrinsic mitochondrial respiratory capacity is decreased in patients with type 2 diabetes (impaired glucose tolerance) (Mogensen et al. 2007), which is completely opposite of what is found in this study, where an impaired glucose tolerance was seen after bed rest (increased area under the curve for insulin and glucose during an OGTT together with a reduced Matsuda index). An increased intrinsic respiratory capacity would tax the electron transport system in each mitochondrion to a higher extent, which would lead to an increased ROS production, which has been linked to impaired glucose tolerance and insulin resistance. Data from Lefort and colleagues support our thoughts above as they reported similar mitochondrial respiratory capacity normalized to muscle weight between lean insulin sensitive and obese insulin‐resistant subjects, with a lower activity of CS in the obese subjects, indicating an increased intrinsic respiratory capacity in this group (Lefort et al. 2010). A positive correlation was seen between intrinsic mitochondrial respiratory capacity and ROS production per mitochondria (CS activity) in this study, which supports our speculations of a higher intrinsic mitochondrial respiratory capacity in glucose intolerant subjects. ROS production was also determined in this study, and whereas no differences were seen in absolute values, ROS production per mitochondria (CS activity) was app. 20% higher after bed rest (*P *=* *0.12). Furthermore a higher content of the antioxidant catalase was found after bed rest, this could account for the lack of change in ROS production, although this is only speculations. No differences in either superoxide dismutase or glutathione peroxidase 1 were observed. It has previously been reported that ROS production increases after 2 weeks of immobilization with no changes in the antioxidant content (catalase, superoxide dismutase and glutathione peroxidase 1) (Gram et al. 2015). Another study reported no changes in antioxidant content (catalase and superoxide dismutase) after 7 days of strict bed rest (Dirks et al. 2016). These data combined with data from the this study could indicate that the antioxidant content is upregulated after a short period of bed rest (3–4 days) with no major increases in ROS production, but that after 1–2 weeks of bedrest/immobilization the antioxidant capacity can no longer match the increases ROS production, which manifests in an increased ROS production with no changes in antioxidant content (ROS removal). Four days of bed rest furthermore lead to a decreased MFN2 protein content, suggesting that the mitochondria are less fused, potentially making them less efficient when it comes to glucose and fatty acid oxidation (Zorzano et al. 2010). MFN2 protein content has previously been reported reduced in patients with type 2 diabetes and obese insulin‐resistant subjects (Bach et al. 2003, 2005) indicating that the mitochondrial dynamics could be important in the development of impaired glucose tolerance and type 2 diabetes. A strong correlation between changes in mitochondrial respiratory capacity and MFN2 normalized to CS activity was seen, supporting a link between mitochondrial dynamics and mitochondrial respiratory capacity.

In summary, an increased intrinsic mitochondrial respiratory capacity was found after 4 days of bed rest, which was accompanied by a reduced glucose tolerance. This is the first time that an increased intrinsic mitochondrial respiratory capacity is reported in humans, when at the same time glucose tolerance is impaired. No difference was seen in ROS production after the 4 days of bed rest, but a strong correlation was seen between changes in intrinsic mitochondrial respiratory capacity and ROS production per mitochondria.

## Conflict of Interest

None declared.
